# Supplementary material for: Ibrutinib Plus RCHOP versus RCHOP Only in Young Patients with Activated B-Cell-like Diffuse Large B-Cell Lymphoma (ABC-DLBCL): A Cost-Effectiveness Analysis
Source: Curr Oncol. 2023 Dec 15;30(12):10488–500. doi: 10.3390/curroncol30120764 (PMC10742773; doi:10.3390/curroncol30120764)
Supplement: Supplementary file 1 [file curroncol-30-00764-s001.zip › curroncol-2707854-supplementary.pdf]

Supplementary Materials

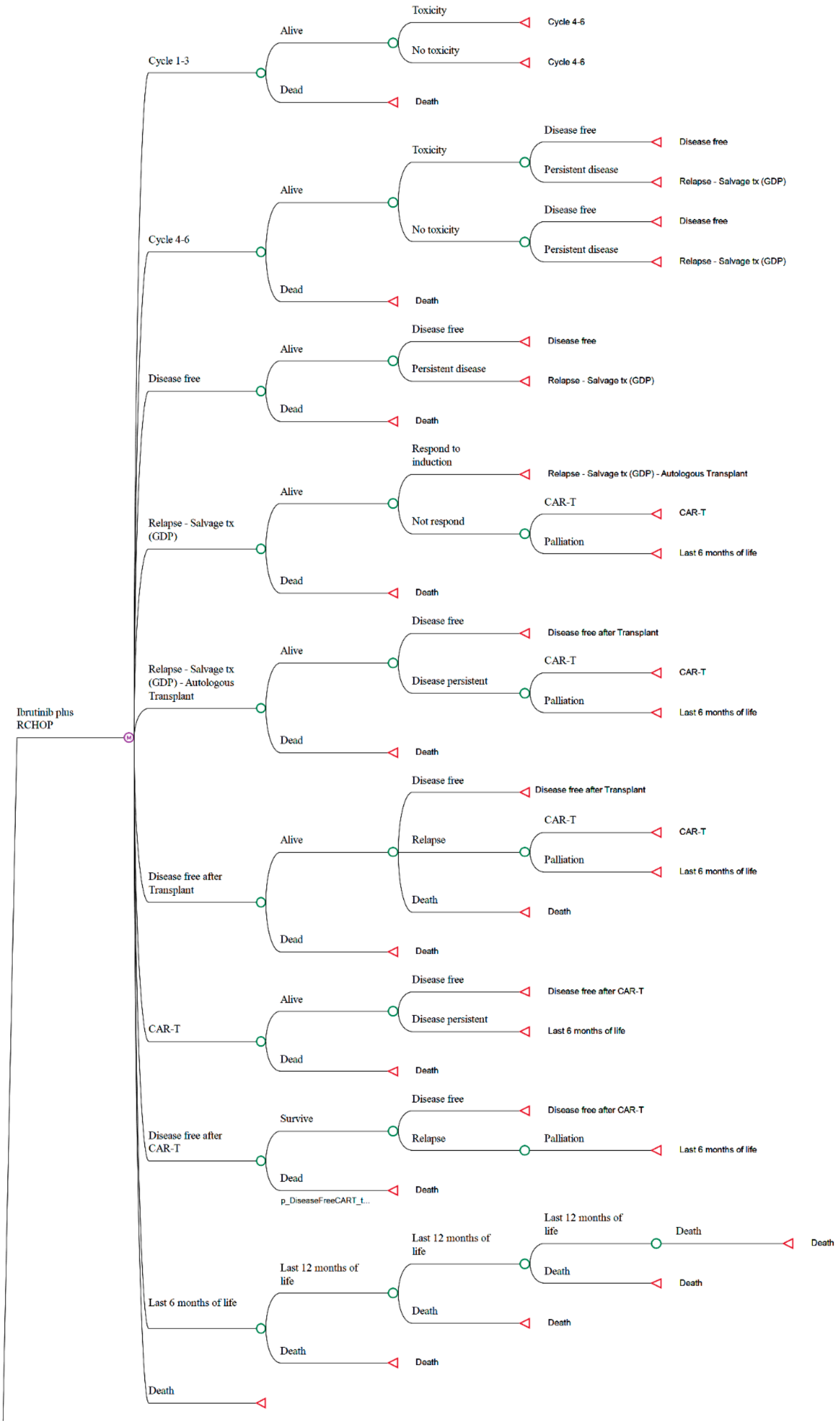

(a)

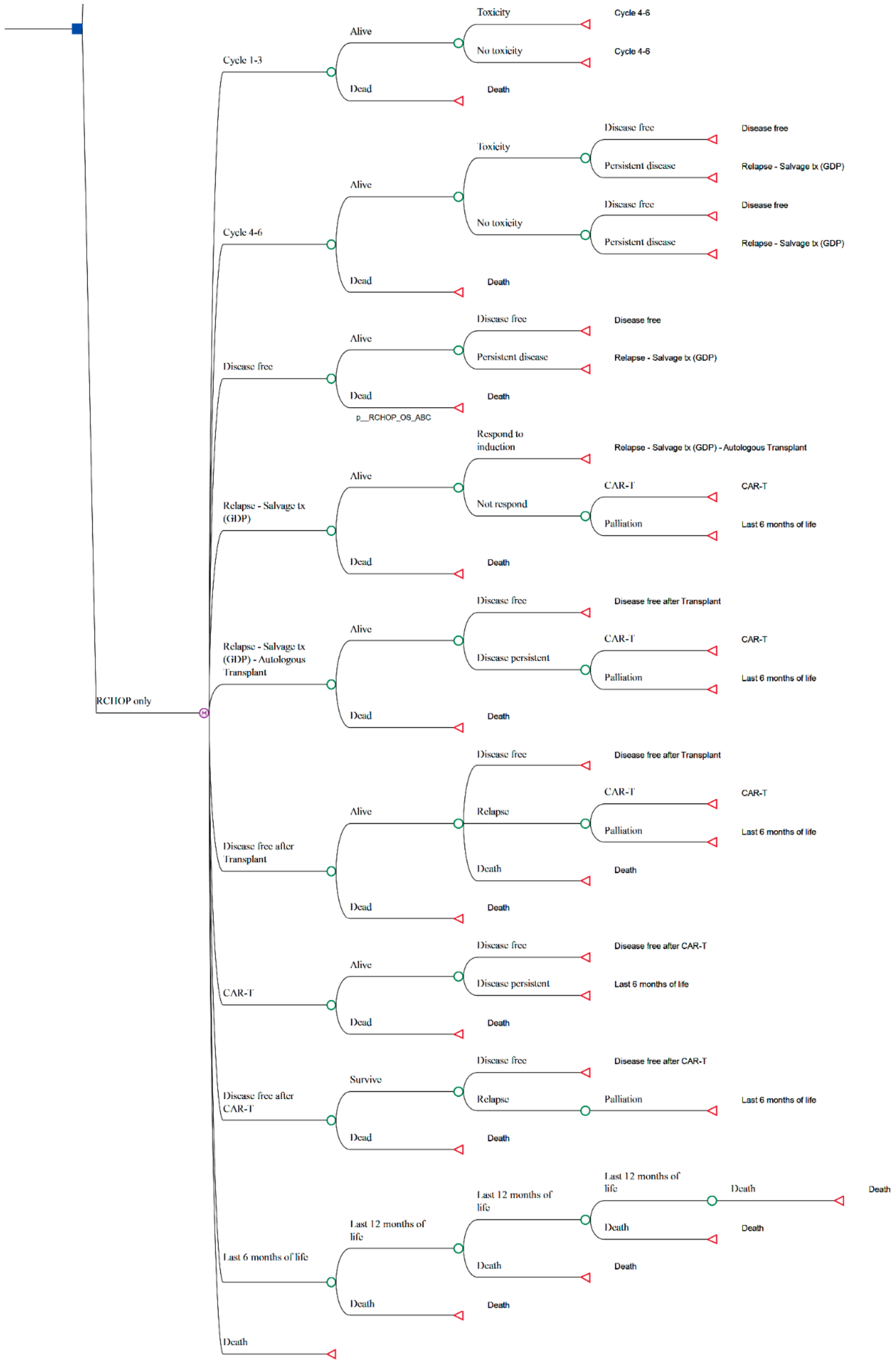

(**b**)

Figure S1. (**a**) Simplified Markov Model (I-RCHOP Arm). (**b**) Simplified Markov Model (RCHOP Arm).

Table S1. Cost estimates used in analysis

Variable	Estimate	Low	High	References
Inclusive of initial treatment, costs of physician visits, nursing costs, supportive medications (G-CSF, anti-emetics), overhead, cancer centre visits, laboratory investigations, PET-CT scans				
RCHOP (total cost for 6 cycles)	42,184	37,275	47,094	[37,63–66]
I-RCHOP (total cost)	109,020	104,114	113,925	[37,63–66]
Disease free period				
Cost per 3 month cycle	361	278	443	[66], Expert Opinion 2021
Salvage Treatment				
GDP (total cost)	1,687	1,324	2,094	[66,67]
Autologous stem cell transplant (total cost, inclusive of GDP)	73,553	49,350	97,756	[66–69]
CAR-T	660,924	654,110	667,738	[66,70]
Palliative Therapy				
Comfort and supportive care in last 12 months of life per 3 month cycle	10136	8926	11346	[69,71], Expert Opinion 2021
Toxicities				
Febrile Neutropenia	9,268	2,915	15,620	[63,66,72]
Nausea	472	64	881	[64,66]
Gamma distributions were assumed for all cost modelling.				

Table S2. Health Utility Data

Utility Description	Utility	Low	High	References
Diagnosis and peri-treatment of DLBCL with RCHOP	0.79	0.693	0.891	[73–76]
Complete Response RCHOP	0.82	0.729	0.913	[73–76]
No Response RCHOP	0.435	0.342	0.539	[73–76]
Diagnosis and peri-treatment of DLBCL with I-RCHOP	0.7505	0.65835	0.84645	[73–76], Expert Opinion 2021
Complete Response I-RCHOP	0.779	0.69255	0.86735	[73–76], Expert Opinion 2021
No Response I-RCHOP	0.41325	0.3249	0.51205	[73–76], Expert Opinion 2021
Hospitalization for febrile neutropenia	0.64	0.512	0.768	[63,77]
GDP Salvage Treatment	0.734	0.5872	0.8808	[78,79]
Receiving autologous stem cell transplant	0.43	0.344	0.516	[69,80]
Post-transplantation	0.7	0.56	0.84	[80]
Complete Response CAR-T	0.73	0.621	0.847	[79,81]
Incurable DLBCL and Palliation	0.435	0.342	0.539	[69,82]
Beta distributions were assumed for all cost modelling.				

References

37. Ministry of Health Ontario. Schedule of Benefits: Physician Services Under the Health Insurance Act. Available online https://www.health.gov.on.ca/en/pro/programs/ohip/sob/physserv/sob_master_20221201.pdf (assessed on 14 October 2022)
63. Lathia, N.; Mittmann, N.; DeAngelis, C.; Knowles, S.; Cheung, M.; Piliotis, E.; Shear, N.; Walker, S. Evaluation of Direct Medical Costs of Hospitalization for Febrile Neutropenia. Cancer 2010, 116, 742–748.
64. Lachaine, J.; Yelle, L.; Kaizer, L.; Dufour, A.; Hopkins, S.; Deuson, R. Chemotherapy-Induced Emesis: Quality of Life and Economic Impact in the Context of Current Practice in Canada. Support. Cancer Ther. 2005, 2, 181–187. https://doi.org/10.3816/SCT.2005.n.011.
65. Best, J.H.; Hornberger, J.; Proctor, S.J.; Omnes, L.F.; Jost, F. Cost-Effectiveness Analysis of Rituximab Combined with Chop for Treatment of Diffuse Large B-Cell Lymphoma. Value Health J. 2005, 8, 462–470. https://doi.org/10.1111/j.1524-4733.2005.00037.x.
66. Ng,P.; Leung, F. Personal Communication. Inpatient Pharmacy, Princess Margaret Cancer Centre: Toronto, ON, Canada, 2021.
67. Moccia, A.A.; Hitz, F.; Hoskins, P.; Klasa, R.; Power, M.M.; Savage, K.J.; Shenkier, T.; Shepherd, J.D.; Slack, G.W.; Song, K.W.; et al. Gemcitabine, Dexamethasone, and Cisplatin (GDP) Is an Effective and Well-Tolerated Salvage Therapy for Re-lapsed/Refractory Diffuse Large B-Cell Lymphoma and Hodgkin Lymphoma. Leuk Lymphoma 2017, 58, 324–332. https://doi.org/10.1080/10428194.2016.1193852.
68. Vijenthira, A.; Kuruvilla, J.; Prica, A. Cost-Effectiveness Analysis of Allogeneic versus Autologous Stem Cell Trans-plant versus Chemo-Immunotherapy for Early Relapse of Follicular Lymphoma within 2 Years of Initial Therapy. Bone Marrow Transplant 2021, 56, 2400–2409. https://doi.org/10.1038/s41409-021-01327-5.
69. Vijenthira, A.; Chan, K.; Cheung, M.C.; Prica, A. Cost-Effectiveness of First-Line Treatment Options for Patients with Ad-vanced-Stage Hodgkin Lymphoma: A Modelling Study. Lancet Haematol 2020, 7, e146–e156. https://doi.org/10.1016/S2352-3026(19)30218-2.
70. Axicabtagene Ciloleucel for Diffuse Large B-Cell Lymphoma: Economic Review Report, 2019. https://www.cadth.ca/sites/default/files/pdf/car-t/ct0002-axicabtagene-ciloleucel-economic-report-redacted.pdf. (accessed on 14 October 2022)
71. Yu, M.; Guerriere, D.N.; Coyte, P.C. Societal Costs of Home and Hospital End-of-Life Care for Palliative Care Pa-tients in Ontario, Canada. Health Soc. Care 2015, 23, 605–618. https://doi.org/10.1111/hsc.12170.
72. Canadian Agency for Drugs and Technologies in Health. Filgrastin (Grastofil - Apotex Inc.). CADTH Canadian Drug Expert Committee Final Recommendation. Available online https://www.cadth.ca/sites/default/files/cdr/complete/SE0446_cdr_complete_Grastofil_March-22-16_e.pdf (assessed on 14 October 2022)
73. Knight, C.; Hind, D.; Brewer, N.; Abbott, V. Rituximab (MabThera) for Aggressive Non-Hodgkin's Lymphoma: A System-atic Review and Economic Evaluation. Health Technol. Assess. 2004, 8, 1–82. https://doi.org/10.3310/hta8370.
74. Oerlemans, S.; Issa, D.E.; van den Broek, E.C.; Nijziel, M.R.; Coebergh, J.W.W.; Huijgens, P.C.; Mols, F.; van de Poll-Franse, L.V. Health-Related Quality of Life and Persistent Symptoms in Relation to (R-)CHOP14, (R-)CHOP21, and Other Therapies among Patients with Diffuse Large B-Cell Lymphoma: Results of the Population-Based PHAROS-Registry. Ann. Hematol. 2014, 93, 1705–1715. https://doi.org/10.1007/s00277-014-2099-8.
75. Wang, H.; Manca, A.; Crouch, S.; Bagguley, T.; Yu, G.; Aas, E.; Howell, D.; Burton, C.; Patmore, R.; Roman, E.; et al. Health-State Utility Values in Diffuse Large B-Cell Lymphoma. Value Health 2018, 21, S74. https://doi.org/10.1016/j.jval.2018.09.433.
76. Golicki, D.; Jaśkowiak, K.; Wójcik, A.; Młyńczak, K.; Dobrowolska, I.; Gawrońska, A.; Basak, G.; Snarski, E.; Hołownia-Voloskova, M.; Jakubczyk, M.; et al. EQ-5D–Derived Health State Utility Values in Hematologic Malignancies: A Catalog of 796 Utilities Based on a Systematic Review. Value Health 2020, 23, 953–968. https://doi.org/10.1016/j.jval.2020.04.1825.
77. Lathia, N.; Isogai, P.K.; Angelis, C.D.; Smith, T.J.; Cheung, M.; Mittmann, N.; Hoch, J.S.; Walker, S. Cost-Effectiveness of Fil-grastim and Pegfilgrastim as Primary Prophylaxis Against Febrile Neutropenia in Lym-phoma Patients. JNCI J. Natl. Cancer Inst. 2013, 105, 1078–1085. https://doi.org/10.1093/jnci/djt182.
78. Cheung, M.C.; Hay, A.E.; Crump, M.; Imrie, K.R.; Song, Y.; Hassan, S.; Risebrough, N.; Sussman, J.; Couban, S.; MacDonald, D.; et al. Gemcitabine/Dexamethasone/Cisplatin vs Cytarabine/Dexamethasone/Cisplatin for Relapsed or Refractory Ag-gressive-Histology Lymphoma: Cost-Utility Analysis of NCIC CTG LY.12. JNCI J. Natl. Cancer Inst. 2015, 107, djv106. https://doi.org/10.1093/jnci/djv106.

79. Ellis, K. Cost-Effectiveness of Chimeric Antigen Receptor T-Cell Therapy for Treating Large B- Cell Lymphoma Patients in Canada. Master’s Thesis, The University of Waterloo, Waterloo, ON, Canada, 2019.
80. Frödin, U.; Börjeson, S.; Lyth, J.; Lotfi, K. A Prospective Evaluation of Patients’ Health-Related Quality of Life dur-ing Au-to-SCT: A 3-Year Follow-Up. Bone Marrow Transplant 2011, 46, 1345–1352. https://doi.org/10.1038/bmt.2010.304.
81. Howell, T.A.; Matza, L.S.; Jun, M.P.; Garcia, J.; Powers, A.; Maloney, D.G. Health State Utilities for Adverse Events Associat-ed with Chimeric Antigen Receptor T-Cell Therapy in Large B-Cell Lymphoma. Pharm. Open 2022, 6, 367–376. https://doi.org/10.1007/s41669-021-00316-0.
82. Wu, H.; Lin, P.; Yang, S.; Zhang, W.; Tao, W. Cost-Utility Analysis of Palliative Care in Patients with Advanced Cancer: A Retrospective Study. BMC Palliat. Care 2021, 20, 126. https://doi.org/10.1186/s12904-021-00816-0.
